# Diversity of Aquatic *Pseudomonas* Species and Their Activity against the Fish Pathogenic Oomycete *Saprolegnia*

**DOI:** 10.1371/journal.pone.0136241

**Published:** 2015-08-28

**Authors:** Yiying Liu, Elzbieta Rzeszutek, Menno van der Voort, Cheng-Hsuan Wu, Even Thoen, Ida Skaar, Vincent Bulone, Pieter C. Dorrestein, Jos M. Raaijmakers, Irene de Bruijn

**Affiliations:** 1 Department of Microbial Ecology, Netherlands Institute of Ecology (NIOO-KNAW), Wageningen, The Netherlands; 2 Laboratory of Phytopathology, Wageningen University, Wageningen, The Netherlands; 3 Division of Glycoscience, School of Biotechnology, Royal Institute of Technology, Stockholm, Sweden; 4 Department of Chemistry and Biochemistry, University of California San Diego, La Jolla, California, United States of America; 5 Department of Chemistry, Boston University, Boston, United States of America; 6 Norwegian Veterinary Institute, Oslo, Norway; 7 Norwegian University of Life Sciences, Oslo, Norway; 8 Skaggs School of Pharmacy and Pharmaceutical Sciences, University of California San Diego, La Jolla, California, United States of America; 9 Center for Marine Biotechnology and Biomedicine, Scripps Institution of Oceanography, University of California San Diego, La Jolla, California, United States of America; 10 Department of Pharmacology, University of California San Diego, La Jolla, California, United States of America; University of Aveiro, PORTUGAL

## Abstract

Emerging fungal and oomycete pathogens are increasingly threatening animals and plants globally. Amongst oomycetes, *Saprolegnia* species adversely affect wild and cultivated populations of amphibians and fish, leading to substantial reductions in biodiversity and food productivity. With the ban of several chemical control measures, new sustainable methods are needed to mitigate *Saprolegnia* infections in aquaculture. Here, PhyloChip-based community analyses showed that the Pseudomonadales, particularly *Pseudomonas* species, represent one of the largest bacterial orders associated with salmon eggs from a commercial hatchery. Among the *Pseudomonas* species isolated from salmon eggs, significantly more biosurfactant producers were retrieved from healthy salmon eggs than from *Saprolegnia*-infected eggs. Subsequent *in vivo* activity bioassays showed that *Pseudomonas* isolate H6 significantly reduced salmon egg mortality caused by *Saprolegnia diclina*. Live colony mass spectrometry showed that strain H6 produces a viscosin-like lipopeptide surfactant. This biosurfactant inhibited growth of *Saprolegnia in vitro*, but no significant protection of salmon eggs against Saprolegniosis was observed. These results indicate that live inocula of aquatic *Pseudomonas* strains, instead of their bioactive compound, can provide new (micro)biological and sustainable means to mitigate oomycete diseases in aquaculture.

## Introduction

Emerging fungal and fungal-like diseases are causing severe ecological disruptions and are recognised as a global threat to biodiversity and food security [1, 2]. For example, *Fusarium solani* is involved in mass mortality of eggs of the endangered sea turtles in Cape Verde [3], and *Batrachochytrium dendrobatidis* and *B*. *salmandrivorans* are causing major amphibian declines globally [4, 5]. Amongst oomycetes, *Aphanomyces* and *Saprolegnia* species are causing significant declines in crayfish, fish and amphibian populations [1, 6–11]. *Saprolegnia* species are the causative agents of Saprolegniosis, a disease characterized by fluffy and filamentous white or grey mycelial patches on fish, fish eggs or amphibians [11]. In aquaculture, *Saprolegnia* species regularly infect freshwater cultured salmonids, including Atlantic salmon and rainbow trout, and non-salmonids like eel, perch, carp and catfish [7, 12]. In Japan, at least 50% annual mortality in Coho salmon due to Saprolegniosis was reported [7, 13, 14]. Also the ‘winter kill’ by *Saprolegnia* species in channel catfish in the USA resulted in a substantial financial loss of approximately $40 million [7].

Formalin is now commonly used to control Saprolegniosis, but is expected to be banned soon due to adverse environmental effects [7]. Several treatments have been tested to prevent Saprolegniosis, such as hydrogen cyanide, Pyceze (bronopol), sea water flushes and NaCl, but none of these measures exerted control to a level similar as obtained with malachite green, a chemical banned due to its carcinogenic properties [7]. Currently, no vaccination is available for Saprolegniosis [8]. Therefore, new sustainable measures are urgently needed. A potential approach to control Saprolegniosis and other emerging diseases involves the application of beneficial microbes. A limited number of bacterial genera and species, including *Aeromonas* and *Pseudomonas*, have been reported as potential anti-pathogen agents in aquaculture, but a comprehensive understanding of the microbiome composition of fish eggs and their protective potential is still limited [15–21].

In previous work, we detected 31,281 bacterial and archaeal operational taxonomic units (OTUs) on salmon eggs from a hatchery by PhyloChip metataxonomic analysis. The highest number of OTUs belonged to Proteobacteria [22]. Based on this large-scale metataxonomic analysis, the diversity of salmon egg-associated Proteobacteria and their functional potential to protect fish eggs against *Saprolegnia* were investigated in this study. We focused specifically on the Pseudomonadales and isolated several *Pseudomonas* strains, assessed their genotypic diversity and tested their inhibitory activity against *Saprolegnia* species both *in vitro* and *in vivo*. Given the zoosporicidal activity of biosurfactants produced by *Pseudomonas* species [23–25], the isolates obtained from salmon eggs were also phenotypically screened for biosurfactant production. For the *Pseudomonas* isolate that provided the best protection against Saprolegniosis on salmon eggs, chemical profiling was performed by Nanospray Desorption ElectroSpray Ionization (NanoDESI) live colony mass spectrometry followed by MS/MS analysis for partial identification of the biosurfactant.

## Materials and Methods

### Isolation of bacteria associated with salmon eggs

Healthy and *Saprolegnia*-infected salmon eggs (*N* = 6 for healthy and *N* = 6 for diseased eggs) and their corresponding incubation water was collected from a commercial hatchery [22]. To release bacteria from the surface of eggs, approximately 30 eggs and 20 ml of incubation water of each sample was transferred into a glass tube, vortexed for one minute, sonicated for one minute and vortexed again for one minute. The total culturable bacteria were isolated and enumerated by plating on 1/10^th^ strength tryptone soya broth (Oxoid) with 15–20 gl^-1^ agar (1/10TSA) supplemented with 100 μg ml^-1^ Delvocid (DSM, Delft, Netherlands) to inhibit fungal growth. *Pseudomonas* strains were isolated and enumerated on semi-selective *Pseudomonas* agar F (PSA, Difco) supplemented with 100 μg ml^-1^ Delvocid, 12.5 μg ml^-1^ chloramphenicol and 50 μg ml^-1^ ampicillin. Both media were incubated at 25°C for 4 days. From each replicate sample and each growth medium, approximately 40 bacterial isolates were randomly selected and stored, which resulted in a total of approximately 900 random bacterial isolates.

### *In vitro* activity and biosurfactant production by the bacterial isolates

All bacterial isolates were tested for activity against *Saprolegnia diclina* strain VS20 and *Saprolegnia parasitica* strain CBS 223.65 (C65) according to Liu *et al*. [22]. Bacteria were spot-inoculated at the edge of plates of 1/5^th^ strength potato dextrose broth (Difco) with 15–20 g l^-1^ agar (1/5PDA) and incubated for 2–4 days at 25°C prior to inoculation of a plug of *Saprolegnia* in the centre of a 1/5PDA plate. Hyphal growth inhibition was monitored for all bacterial isolates during incubation for 4–5 days at 18°C. All bacterial isolates were also screened for biosurfactant production by the drop collapse assay according to the method described by de Bruijn *et al*. [23].

### Identification and phylogeny of bacterial isolates

*Pseudomonas* isolates inhibiting *Saprolegnia* hyphal growth and/or producing biosurfactants were subjected to BOX-PCR fingerprinting using primer BOX A1R [26, 27]. Representative isolates from the BOX groups with at least 4 isolates for diseased or for healthy salmon egg samples were chosen for phylogenetic analysis. Therefore, a total of 27 representative isolates (S1 Table) were selected and identified by 16S rRNA sequencing. Phylogenetic analyses, including the 16S rRNA sequences of 29 known reference strains [28], was performed according to Liu *et al*. [22]. The evolutionary distances were calculated using the Kimura 2-parameter method [29].

### *In vivo* bioassays to test disease suppression by Proteobacteria

The representative isolates were chosen for activity testing in salmon egg bioassays (S1 Table). From the shared representative isolates, *Pseudomonas* isolates S1 and S2, which belonged to the largest shared BOX group and originated from both healthy and diseased salmon eggs were selected. Collectively, a total of 11 representative isolates were selected for activity testing in salmon egg bioassays (S1 Table).

The experimental set-up of the *in vivo* bioassays was similar to that described by Liu *et al*. [22]. For bioassay 1 conducted in Norway in 2012, salmon eggs used in this bioassay were 385 degree-days at the day of shipment. Each treatment was conducted in a separate incubation unit containing three perforated cups with 30 live salmon eggs per cup. The 11 representative isolates described above were pre-grown on PSA for 2 days at 25°C, washed with sterile de-mineralized water and added to each salmon egg incubation unit to a final cell density of 10^8^ CFU ml^-1^. *S*. *diclina* 765F3 and bacteria were added to the incubation units with 2.5 litre of de-chlorinated Norwegian tap water on day 0 (0 day post inoculation) and egg mortality was scored on day 6 (6 days post inoculation, dpi) and expressed as a percentage of the total number of salmon eggs. To further study the activity spectrum of the *Pseudomonas* isolates, a similar experiment was conducted with *S*. *parasitica* 762F4 [22]. To check if the bacterial isolates alone were pathogenic to salmon eggs, a similar bioassay as described above was conducted with the bacterial inoculum only. Also here, the percentage of egg mortality was determined at 6 dpi.

For bioassay 2 conducted in The Netherlands in 2014, the experimental design was adjusted to monitor the disease progress over a longer period of time. Therefore, the salmon eggs were ‘younger’ than those in bioassay 1, i.e. 321 degree-days at the day of shipment. Each treatment was conducted in two separate incubation units; each incubation unit contained three perforated cups with 51±2 live salmon eggs per cup. Spontaneous rifampicin resistant mutants of the *Pseudomonas* isolates were generated to allow monitoring of their population dynamics. The *Pseudomonas* isolates were added to each salmon egg incubation unit filled with 2 litres of well water [22] to the final cell density of 10^7^ CFU ml^-1^. *S*. *diclina* 1152F4 [22] was used in bioassay 2. Salmon eggs not treated with the bacterial isolates or exposed to *S*. *diclina* only served as the controls. The egg incubation water of each incubation unit was sampled on day 1, day 4 (0 dpi) and day 24 (20 dpi) and dilution-plated on PSA+rifampicin to determine the density of the introduced isolates in the water. Colonization of the applied *Pseudomonas* isolates on the salmon egg surface was determined by rolling one to two eggs from each cup on PSA+rifampicin at 0 and 20 dpi as described by Liu *et al*. [22]. The percentage of salmon eggs to which hyphae of *S*. *diclina* were attached was determined at 20 dpi according to Liu *et al*. [22].

*S*. *diclina* 765F3 from the healthy sample and *S*. *diclina* 1152F4 from the diseased sample were used as pathogen source in the bioassay 1 in 2012 and bioassay 2 in 2014, respectively. These two *S*. *diclina* isolates showed similar pathogenicity on salmon eggs in 2012 [22].

### Nucleotide sequence accession numbers

The 16S rRNA sequences of *Pseudomonas* strains D1, D2, D3, S1, S2, H1, H2, H3, H4, H5, H6 and S3-S18 have been deposited in GenBank under accession numbers KP890304-KP890314 and KT223371-KT223386, respectively.

### Live colony NanoDESI MS/MS data acquisition and molecular networking

The most antagonistic *Pseudomonas* isolate H6 was subjected to chemical profiling by Nanospray Desorption ElectroSpray Ionization (NanoDESI) live colony mass spectrometry as described previously [30, 31]. *P*. *fluorescens* SS101 and *P*. *fluorescens* SBW25 [30] were used as references. *Pseudomonas* strains were cultured in Luria-Bertani broth by shaking overnight at 28°C. For *Pseudomonas* H6, four cultures (0.5 μl each) were spot-inoculated on ISP2 agar and incubated for 48 hours at 30°C [30]. *P*. *fluorescens* SS101 and *P*. *fluorescens* SBW25 were streaked on 1/5^th^ strength NBY agar (1 gl^-1^ glucose, 1.6 gl^-1^ nutrient broth, 0.4 gl^-1^ yeast extract and 15 gl^-1^ agar) and cultured for 48 hours at 25°C. Data collection with a data-dependent MS/MS method was conducted on a hybrid 6.4T LTQ-FT (Thermo Electron) mass spectrometer according to Nguyen *et al*. [30].

The MS/MS data of strains *Pseudomonas* H6, *P*. *fluorescens* SS101 and *P*. *fluorescens* SBW25 were combined with data obtained by Nguyen *et al*. (2013) [30] for 18 other Pseudomonads and 42 Bacilli. Metabolic networks were generated by clustering as described previously [32] using the GNPS website (http://gnps.ucsd.edu/). Algorithms were the same as described by Nguyen *et al*. [30]. Networks were visualized using Cytoscape (v 3.1.1). The two plugins used for aiding data visualization and sequence tagging were described previously by Nguyen *et al*. [30].

### HPLC analysis

*Pseudomonas* H6, *P*. *fluorescens* SS101 and *P*. *fluorescens* SBW25 were pre-grown on PSA for 3–6 days at 25°C. Extraction of biosurfactants was conducted according to De Souza *et al*. [24]. After lyophilization, the biosurfactant was dissolved in Milli-Q water. Reversed-Phase High Performance Liquid Chromatography (RP-HPLC) analysis was performed by injection of 100 μl sample on a Waters 996 HPLC equipped with a Symmetry C18 Column (100 Å, 5 μm, 3.9 mm X 150 mm, Waters) as described previously [33]. Samples were analysed at a flow rate of 0.5 ml/min for 50 min in an isocratic mobile phase of 45:40:15 acetonitrile:methanol:Milli-Q water with 0.1% (v/v) trifluoroacetic acid.

### *In vitro* inhibitory activity of biosurfactants against *Saprolegnia* hyphal growth

The biosurfactant of *Pseudomonas* H6 was tested for activity against hyphae of *S*. *diclina* 1152F4 and *S*. *parasitica* C65 by adding the biosurfactant to 1 ml 1/5^th^ strength potato dextrose broth (1/5PDB) in 24-well cell-culture plates (Greiner Bio-One, Kremsmünster, Austria) to final concentrations of 15, 40, 100, 200 μg ml^-1^, respectively. The lipopeptide surfactant massetolide A produced by *P*. *fluorescens* SS101 was used as a standard. An agar plug of pre-grown *S*. *diclina* 1152F4 or *S*. *parasitica* C65 of approximately 0.2 cm^2^ was added to each well and incubated at 25°C. Hyphal growth inhibition was scored after 3 days for *S*. *diclina* 1152F4 or 2 days for *S*. *parasitica* C65. Morphological abnormalities were monitored under an Olympus SZX12 stereomicroscope and image capture was accomplished using a Zeiss AxioCam MRc 5 camera with Zeiss AxioVision software (AxioVs40 V 4.8.2.0). ImageJ 1.47v [34] was used to measure the diameter of 10 hyphae in each treatment.

### *In vivo* effects of biosurfactants on Saprolegniosis

The salmon eggs had been incubated at 0.92°C on average at AquaGen AS (Trondheim, Norway) and their age was 135 degree-days at the day of shipment. The set-up of the salmon egg bioassay was similar to that of bioassay 2 described above. The biosurfactants from *Pseudomonas* H6 and SS101 were tested at concentrations of 15±3 μg ml^-1^ and 40±8 μg ml^-1^. Malachite green (2.5±0.5 μg ml^-1^ (ppm)) was used as a chemical reference. All treatments were performed with three separate incubation units (biological replicates), except for the treatments without *S*. *diclina* 1152F4 where one incubation unit with three incubation cups (technical reps) was used; each cup contained 51±2 live salmon eggs. Application of biosurfactants or malachite green was conducted every 2–3 days by reducing the water level to 600±100 ml and exposing the salmon eggs to the corresponding chemicals for 90–120 min with aeration. Afterwards, the treated water was removed. Each incubation unit was rinsed by 100–200 ml fresh well water and finally 2 litres of fresh well water was added. The percentage of hyphal attachment of *S*. *diclina* to the salmon eggs was determined on 18 dpi.

## Results and Discussion

### PhyloChip-based community profiling

**Fig 1:**
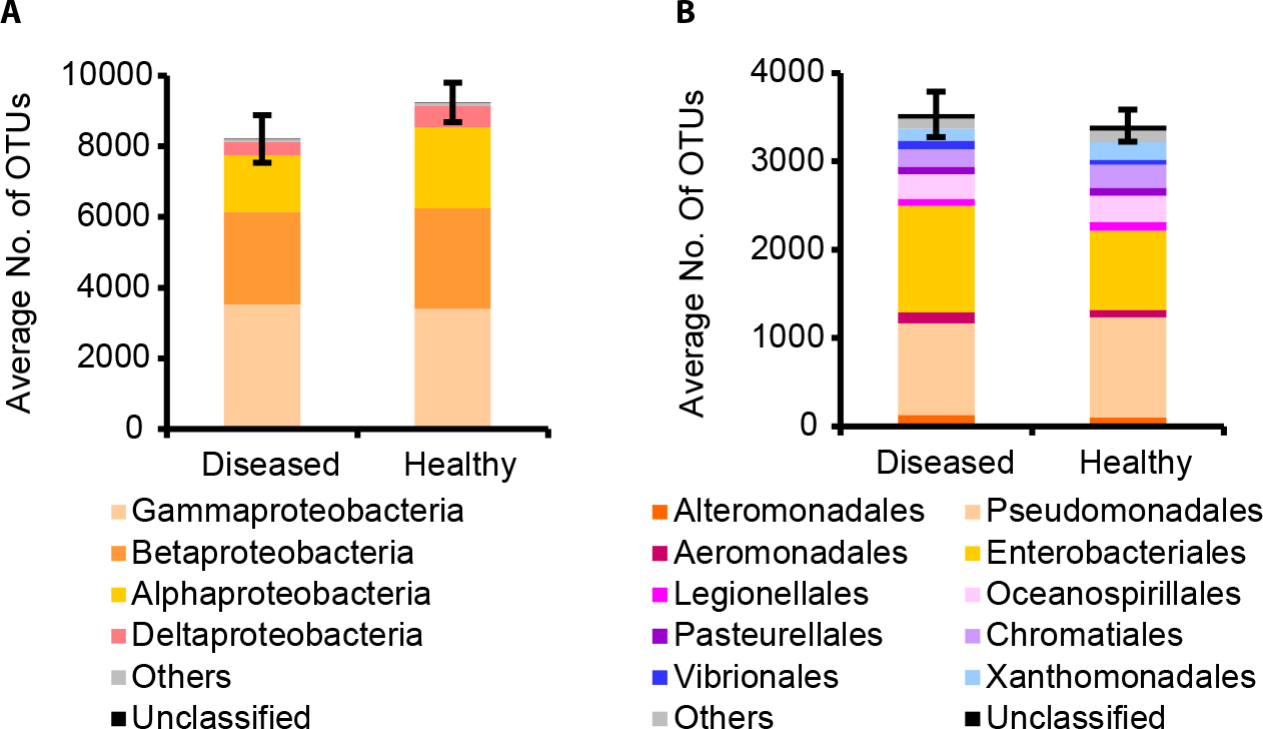
Proteobacteria (A) and Gammaproteobacteria (B) community analyses of diseased and healthy salmon eggs by PhyloChip-based analyses. Salmon eggs were sampled from a commercial hatchery [22]. Indicated is the average number of OTUs at different taxonomic levels: the phylum Proteobacteria (A) and the class Gammaproteobacteria (B). Error bars represent S.E.M. (*N* = 6).

Previously we analysed the bacterial community compositions of healthy salmon eggs and *Saprolegnia*-infected salmon eggs (referred to as diseased salmon eggs) by PhyloChip-based profiling [22]. Healthy eggs harboured more OTUs belonging to the Proteobacteria than diseased eggs [22]. Amongst the phylum Proteobacteria, the class Gammaproteobacteria was most represented (Fig 1A). Within the Gammaproteobacteria, most OTUs belonged to the orders Enterobacteriales and Pseudomonadales (Fig 1B), accounting for 6.57% and 6.71% of the total bacterial OTUs detected on average on salmon eggs, respectively. The majority of OTUs of the family Pseudomonadaceae belonged to *Pseudomonas*, representing 935 OTUs on average. No significant differences were found in the number of *Pseudomonas* OTUs between diseased and healthy salmon eggs. Given that *Pseudomonas* species (Pseudomonadales) are considered as potential aquaculture probiotics [35], a more in-depth analysis was conducted here to unravel the genotypic and functional diversity of *Pseudomonas* species associated with healthy and diseased salmon eggs (S1 Fig).

### Isolation, *in vitro* activity and phylogeny of aquatic *Pseudomonas*

**Fig 2:**
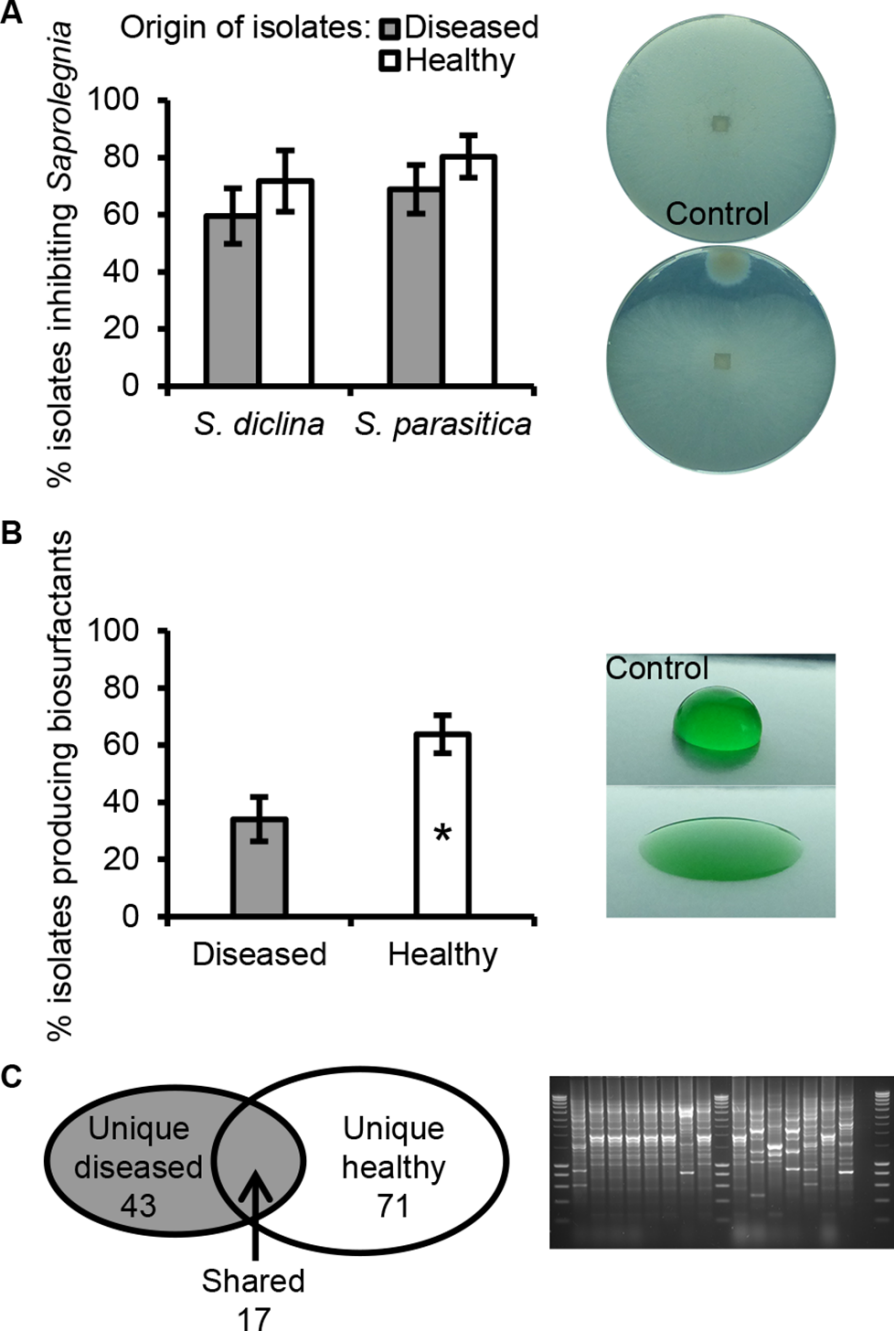
Phenotypic and genotypic analysis of *Pseudomonas* isolates. The isolates were obtained from healthy salmon egg samples and salmon eggs infected with *Saprolegnia*. (A) Mean percentage of the number of bacterial isolates that were inhibitory to hyphal growth of *Saprolegnia diclina* VS20 and *Saprolegnia parasitica* CBS 223.65, tested by observation of an inhibition halo around the bacterial colony (right panel insert). (B) Mean percentage of the number of bacterial isolates that produce biosurfactants based on the drop collapse assay (right panel insert). Error bars represent the S.E.M. (*N* = 6). The asterisk indicates a statistically significant difference (*P*<0.05, Student’s *t*-test). (C) Genotypic BOX-PCR grouping of bacterial isolates that inhibiting *Saprolegnia* hyphal growth and/or producing biosurfactants. The Venn diagram (left panel insert) shows the total number of BOX groups obtained for isolates from either diseased, healthy or both samples. An example of the DNA profiles obtained by BOX-PCR is shown in agarose gel (right panel insert).

The bacteria attached to the salmon eggs were released by vortexing and sonication in the incubation water from the hatchery. The total count of culturable aerobic bacteria was approximately 3X10^7^ CFU ml^-1^ for both healthy and diseased salmon eggs (S2 Fig). Amongst 440 randomly selected isolates, 7% and 13% inhibited hyphal growth of *Saprolegnia diclina* VS20 and *Saprolegnia parasitica* C65, respectively. Drop collapse assays further revealed that 3% of the 1/10TSA isolates produced biosurfactants under the experimental conditions tested. For both the healthy and diseased salmon egg samples, the putative *Pseudomonas* population density enumerated on semi-selective PSA medium was approximately 10^6^ CFU ml^-1^ (S2 Fig). Amongst 465 randomly selected *Pseudomonas* isolates, 60–69% of the isolates from the diseased salmon eggs and 72–80% of the isolates from the healthy salmon eggs inhibited hyphal growth of *S*. *diclina* VS20 and/or *S*. *parasitica* C65. No statistically significant difference was observed between diseased and healthy salmon eggs in the percentage of isolates with *in vitro* growth-inhibiting activities (Fig 2A). However, based on the results obtained in drop collapse assays, healthy eggs harboured a significantly higher frequency (64±7%) of biosurfactant-producing *Pseudomonas* isolates than diseased salmon eggs (34±8%) (Fig 2B). A marine biosurfactant-producing *Lactobacillus pentosus* provided protection for the crustacean *Artemia* against pathogenic *Vibrio alginolyticus*, suggesting a potential role of biosurfactants in disease suppression [36].

**Fig 3:**
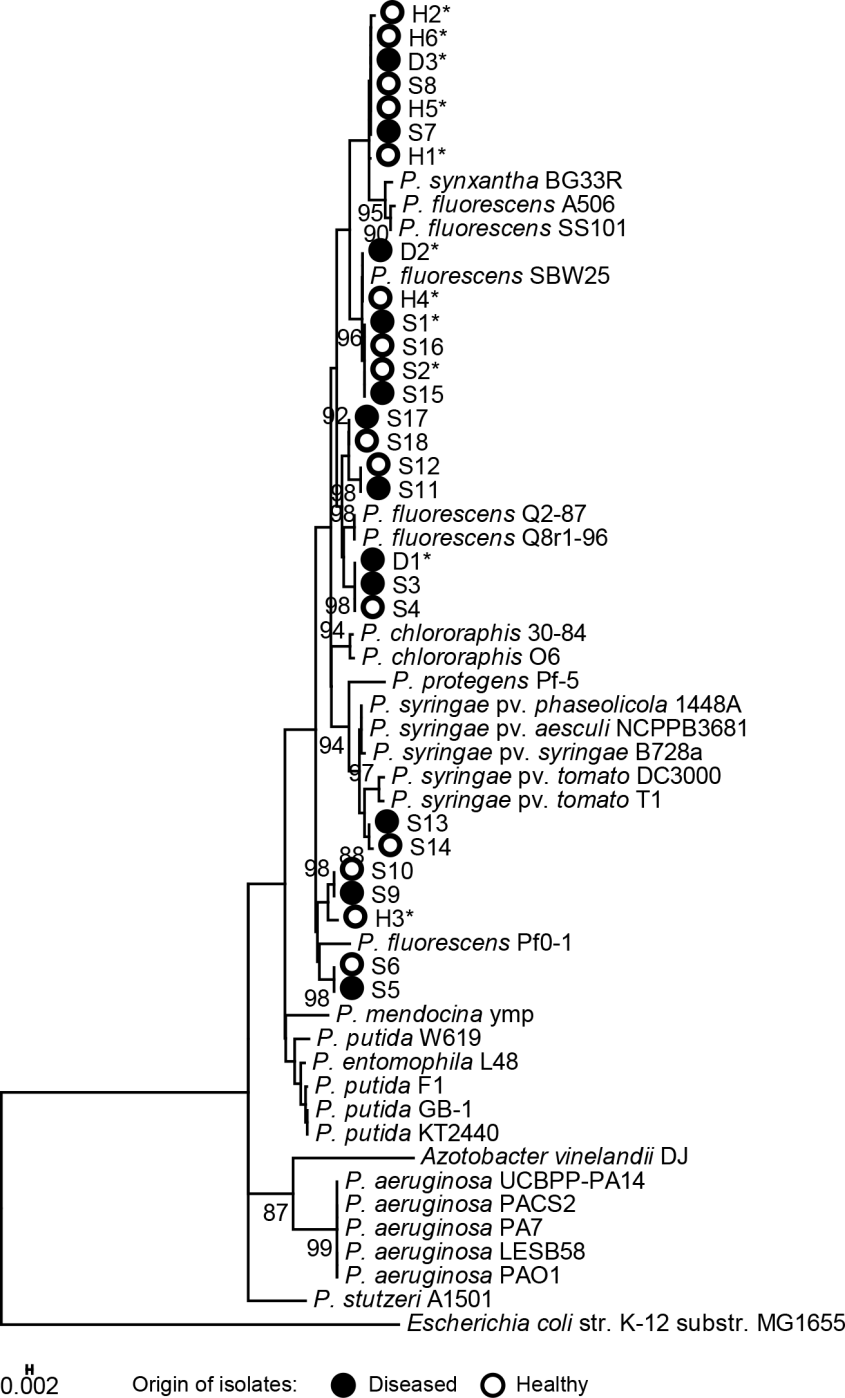
Phylogenetic tree of 16S rRNA sequences of *Pseudomonas* strains representative of 18 BOX-PCR groups. The 16S rRNA sequences were approximately 960 bp. The BOX-PCR groups were identified among the *Pseudomonas* isolates from healthy and diseased salmon eggs (S1 Table). A total of 29 reference *Pseudomonas* species/strains were included to delineate the 27 aquatic *Pseudomonas* strains obtained in this study. Bootstrap values at the nodes are based on 1000 replications. Only those branch values higher than 80% are shown. Asterisks indicate the isolates selected for salmon egg bioassays.

Genotypic profiling by BOX-PCR of the *Pseudomonas* isolates that inhibited *Saprolegnia* hyphal growth *in vitro* and/or produced biosurfactants, resulted in 131 BOX groups with 43 and 71 unique groups from diseased (D) or healthy (H) salmon egg samples, respectively, and 17 groups with isolates found in both diseased and healthy samples (referred to as ‘shared’ isolates (S)) (Fig 2C). Representative isolates from BOX groups that consisted of at least 4 isolates unique for diseased (D) or healthy (H) samples, as well as 18 isolates from the 9 ‘shared’ (S) BOX groups (S1 Table) were subjected to phylogenetic analyses. No distinct differences were observed in the 16S rRNA-based phylogenetic delineation of *Pseudomonas* isolates from diseased or healthy salmon eggs. Most of the isolates belonged to the *P*. *fluorescens* clade, including the isolates from the shared BOX groups, except isolate S13 and S14 that clustered with *P*. *syringae* (Fig 3).

### Bioactivity of aquatic *Pseudomonas in vivo*

**Fig 4:**
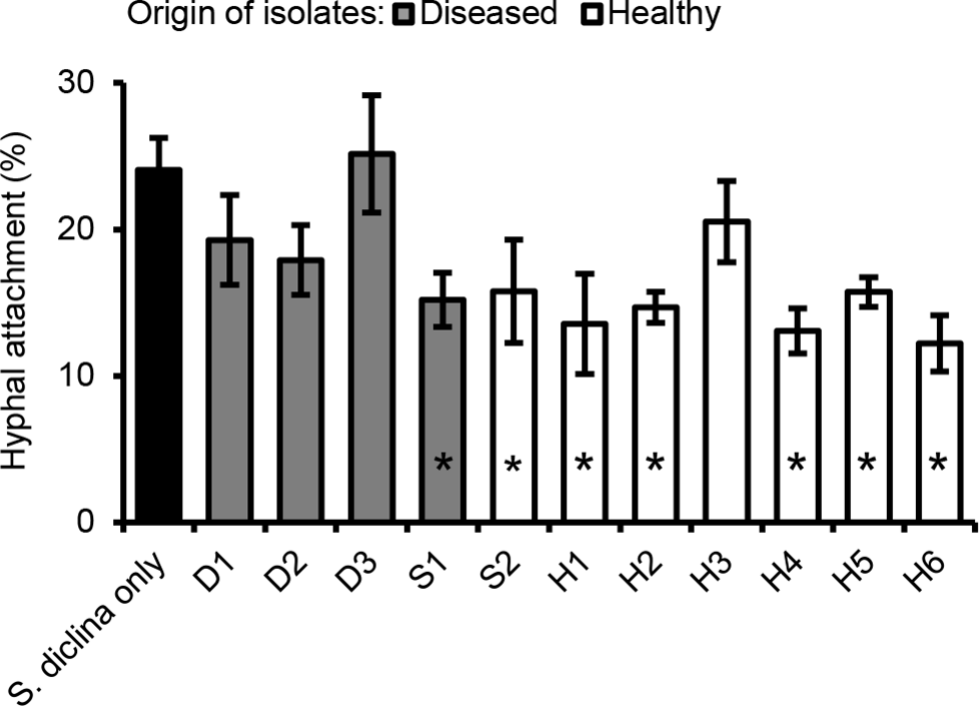
*In vivo* activity of 11 *Pseudomonas* strains against *S*. *diclina* 1152F4. In bioassay 2, the mean percentage of eggs to which of *S*. *diclina* 1152F4 hyphae attached was determined at 20 days post inoculation (dpi) [22]. Bacterial strains were introduced at an initial cell density of 10^7^ CFU ml^-1^. The incubation temperature was 5–7°C. Error bars represent S.E.M. (*N* = 6). Asterisks indicate statistically significant differences with the control, *S*. *diclina* only, based on a one-way analysis of variance and *post hoc* LSD analysis (*P*<0.05).

When *Pseudomonas* isolates were applied at an initial density of 10^7^ CFU ml^-1^ to the incubation water *in vivo*, seven out of eleven *Pseudomonas* isolates significantly reduced hyphal attachment of *S*. *diclina* to salmon eggs (Fig 4). Strains originally isolated from healthy salmon egg samples showed a better control efficacy than those originally isolated from diseased salmon egg samples (Fig 4), suggesting that the healthy salmon eggs harbour *Pseudomonas* strains with stronger activity against *Saprolegnia*. The bacterial density in the incubation water decreased from 10^6^−10^7^ CFU ml^-1^ on 0 dpi to 10^3^−10^5^ CFU ml^-1^ on 20 dpi. When tested against another *S*. *diclina* isolate and *S*. *parasitica* isolate under different temperatures and bacterial densities, only *Pseudomonas* strain H6 showed the most consistent activity against *Saprolegnia* in all cases (S3 and S4A Figs). Hence, strain H6 was selected for further characterization.

### Chemical profiling of aquatic *Pseudomonas* strain H6

**Fig 5:**
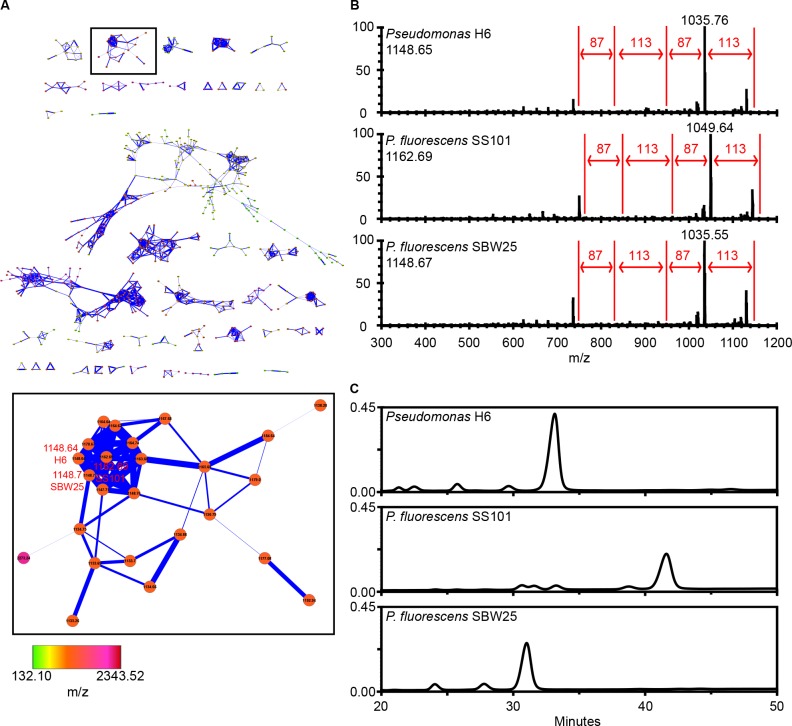
Live colony mass spectrometry and MS/MS networking. (A) Molecular network of *Pseudomonas* H6, *P*. *fluorescens* SS101, *P*. *fluorescens* SBW25 and 18 other Pseudomonads and 42 Bacilli [30]. The viscosin-like cluster was boxed and enlarged. (B) Selection of nodes of *Pseudomonas* H6, *P*. *fluorescens* SS101 and SBW25 for MS/MS raw spectra analyses and generation of amino acid sequence tag. The sequence tag is created by analysing the mass shifts between adjacent ions in the MS/MS spectra, which are corresponding to the mass of an amino acid. The value of the parent mass ions for *Pseudomonas* H6 and *P*. *fluorescens* SBW25 in Fig 5B is not completely matching to the parent mass ion as indicated in Fig 5A, because during network analyses a mass tolerance setting of 0.3 Da was used. (C) Reversed-phase HPLC chromatograms of cell-free culture extracts of lipopeptide surfactants from *Pseudomonas* H6, *P*. *fluorescens* SS101 and SBW25.

To elucidate which compounds *Pseudomonas* strain H6 produces, live colony mass spectrometry and MS/MS analyses were performed with two phylogenetically related strains *P*. *fluorescens* SS101 and SBW25 as references. A Cytoscape network with spectra of in total 21 Pseudomonads and 42 Bacilli strains [30] showed that *Pseudomonas* H6 produces a predominant compound with a parent mass-to-charge ratio of *m/z* 1148.65, which clustered together with the lipopeptides massetolide A (*m/z* 1162.69) and viscosin (*m/z* 1148.67) produced by strains SS101 and SBW25, respectively (Fig 5A). Further examination of the raw MS/MS data showed that mass shifts could be linked to specific amino acids, which created identical sequence tags of 87-113-87-113 Da (Ser-Leu/Ile-Ser-Leu/Ile) for the lipopeptide surfactants produced by *Pseudomonas* H6, *P*. *fluorescens* SS101 and SBW25 (Fig 5B) [30]. These results suggest that *Pseudomonas* H6 produces a lipopeptide surfactant with a peptide moiety that is, most likely, structurally similar to that of massetolide A and viscosin. RP-HPLC analysis of the lipopeptide surfactants extracted from these three strains revealed a difference in retention time of the biosurfactants from *Pseudomonas* H6, *P*. *fluorescens* SS101 and SBW25 (Fig 5C), suggesting that the lipopeptide biosurfactant produced by *Pseudomonas* H6 is structurally not identical to massetolide A or viscosin. The small shift in retention could also be due to a structural difference in the lipid moiety.

### Activity profiling of lipopeptide surfactant from aquatic *Pseudomonas* strain H6

**Fig 6:**
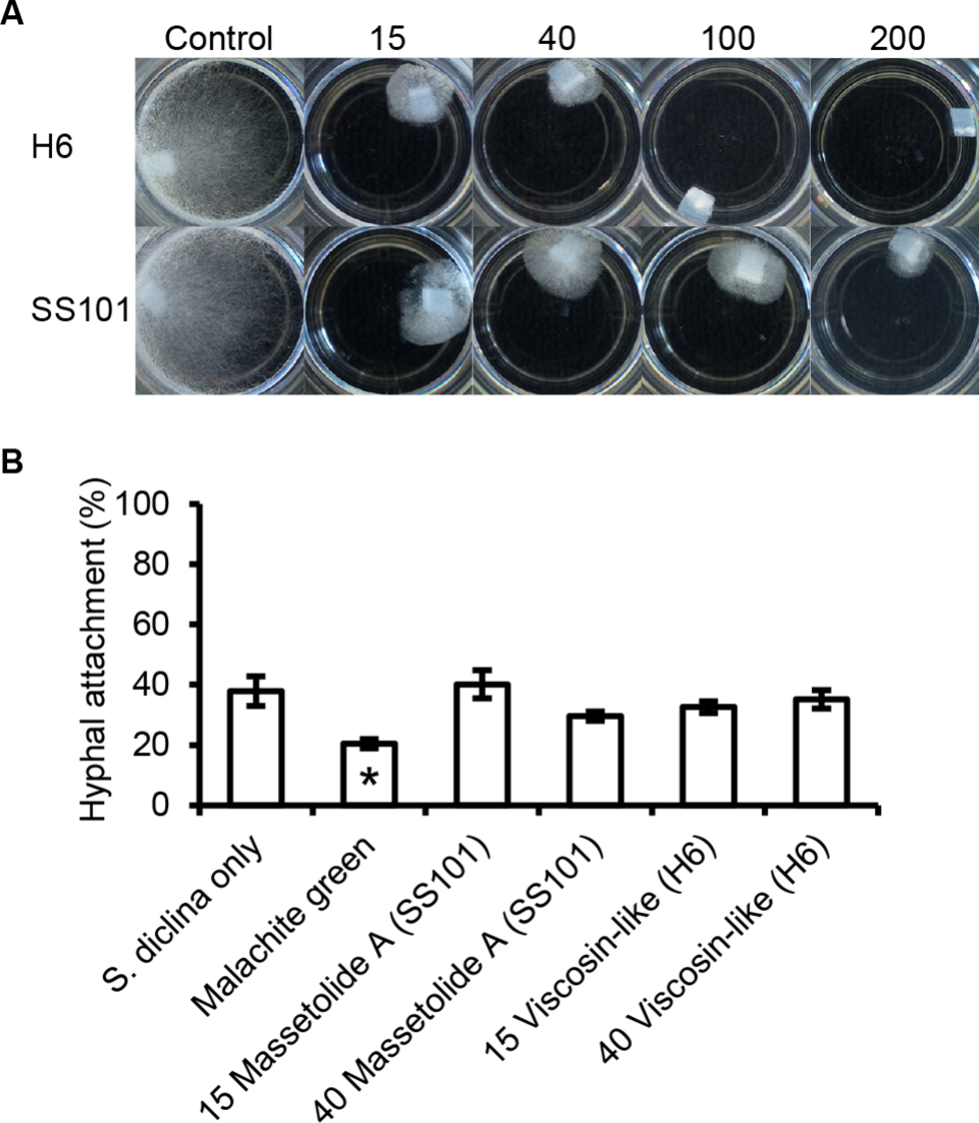
Effect of biosurfactants from *Pseudomonas* H6 and *P*. *fluorescens* SS101 on *S*. *diclina* 1152F4. (A) *S*. *diclina* 1152F4 hyphal plugs were incubated for 3 days in 1/5PDB supplemented with biosurfactants from *Pseudomonas* H6 or *P*. *fluorescens* SS101 (massetolide A) at concentrations ranging from 15 to 200 μg ml^-1^. (B) The activity of the biosurfactants against *S*. *diclina* under *in vivo* condition was assessed by determining the mean percentage of salmon eggs to which *S*. *diclina* 1152F4 hyphae were attached at 18 dpi. Salmon eggs were treated with biosurfactants from *Pseudomonas* H6 and *P*. *fluorescens* SS101 at 15±3 μg ml^-1^ and 40±8 μg ml^-1^. Eggs exposed to *S*. *diclina* only were used as negative control. Eggs treated with 2.5±0.5 μg ml^-1^ (ppm) malachite green and *S*. *diclina* were used as the chemical reference. Error bars represent S.E.M. (*N* = 9). The asterisk indicates a statistically significant difference from the negative control (*S*. *diclina* only) based on one-way analysis of variance and *post hoc* LSD analysis (*P*<0.05).

**Fig 7:**
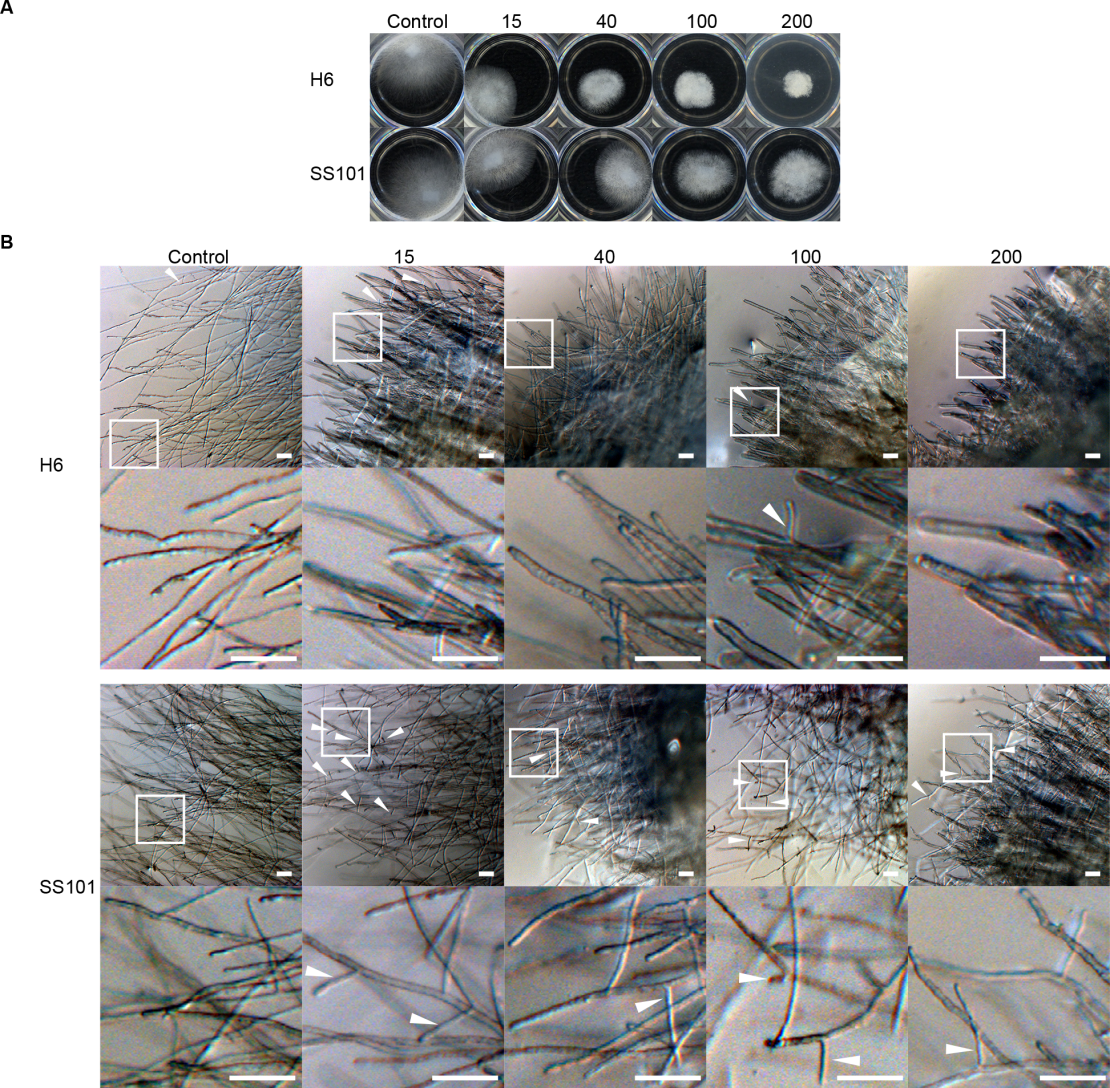
Effect of biosurfactants from *Pseudomonas* H6 and *P*. *fluorescens* SS101 on *S*. *parasitica* CBS 223.65. The effect was according to hyphal growth (A) and hyphal morphology (B). *S*. *parasitica* hyphal plugs were grown for 48 hours in 1/5PDB supplemented with biosurfactants from *Pseudomonas* H6 or *P*. *fluorescens* SS101 (massetolide A) at concentrations ranging from 15 to 200 μg ml^-1^. The bottom row shows the enlargement of the area indicated in the top row. White arrows indicate branching hyphae. The scale bars in the pictures of panel B represent 100 μm.

We tested the effect of the purified lipopeptide surfactants of *Pseudomonas* H6 and *P*. *fluorescens* SS101 on hyphal growth of *S*. *diclina* 1152F4 (Fig 6A). Both surfactants showed growth-inhibitory activity at 15 μg ml^-1^, the lowest concentration tested. Hyphal growth was almost completely inhibited at 100 μg ml^-1^ for the biosurfactant of *Pseudomonas* H6 and at 200 μg ml^-1^ for massetolide A of *P*. *fluorescens* SS101 (Fig 6A). The biosurfactant of H6 also showed a stronger activity compared to massetolide A against hyphal growth of *S*. *parasitica* (Fig 7A). Lipopeptide surfactants are known to cause hyphal swelling, hyphal branching, zoospore lysis and inhibition of cyst germination of plant pathogenic oomycetes [23, 37, 38]. When we investigated the effect of the lipopeptide surfactants of strains H6 and SS101 microscopically, the diameter of *S*. *parasitica* hyphae grown in the presence of biosurfactants was larger (S2 Table) and exhibited a higher number of branches compared to the control. This phenotypic effect intensified with increasing biosurfactant concentrations; furthermore, massetolide A induces more hyphal branching than the *Pseudomonas* H6 biosurfactant (Fig 7B).

The lipopeptide surfactants from *Pseudomonas* H6 and *P*. *fluorescens* SS101 were tested in an *in vivo* bioassay at concentrations of 15±3 μg ml^-1^ and 40±8 μg ml^-1^ of incubation water. Because the set-up of the *in vivo* bioassays involved large volumes and the yield of biosurfactants was relatively low, higher biosurfactant concentrations were not tested. At the concentrations tested, no significant reductions in attachment of *S*. *diclina* hyphae to salmon eggs were found, whereas the chemical control (malachite green) did significantly reduce hyphal attachment (Fig 6B). Although the lipopeptide surfactants were applied every 2–3 days for a duration of 90–120 min each time, instability (e.g. degradation) and/or a too short exposure time may have caused a lack of *in vivo* activity. Biosurfactants have been shown to be degraded in aquatic systems and non-sterile soil [39–41] and the same may have occurred in the salmon egg incubation units. The fate of the biosurfactants could not be analysed by RP-HPLC analysis, since the applied concentrations were below the detection limit. Although the biosurfactants alone did not affect hyphal attachment by *Saprolegnia in vivo*, this does not exclude that these compounds may play a role in the activity of the producing bacterial strain, an aspect that remains to be further investigated. Lipopeptide surfactants are well-known for their role in biofilm formation [23, 37] and may have enabled *Pseudomonas* H6 to colonize the salmon egg surface to form a protective biofilm that avoids hyphal attachment by *Saprolegnia*. Also the biosurfactant produced by *Lactobacillus pentosus* was suggested to facilitate the adhesion to the *Artemia* gut, thereby excluding the colonization of pathogenic *Vibrio alginolyticus* [36]. The bacterial biofilm provides an ecological niche in which the bacterial cells produce a protective extracellular matrix that is beneficial to their growth and development by excluding other microbes [35]. Whether the biosurfactants are actually produced in the egg incubation units or on the egg surfaces by the introduced *Pseudomonas* strains is not known. Recently, Song and colleagues [42] showed that massetolide A production by *P*. *fluorescens* SS101 increased at lower temperatures, indicating that the low temperatures (5–10°C) used in the salmon egg assays may be favourable for biosurfactant production. To provide more evidence for a role of the lipopeptide surfactant of *Pseudomonas* H6 in *in situ* production and protection against Saprolegniosis, site-directed mutagenesis of the biosynthetic genes should be performed in future studies, followed by *in vivo* bioassays where gene transcriptional analyses are conducted and activities of surfactant-deficient mutants are compared to those of the wild type strain H6. Additionally, even though *P*. *fluorescens* is recognized as a beneficial microbe against *Saprolegnia* [20], is it also known to be pathogenic to a wide range of fish species, mostly to carps [43] but also to salmonids like rainbow trout (*Oncorhynchus mykiss*) [43] and Chinook salmon (*Oncorhynchus tshawytscha*) [44]. For some of the *Pseudomonas* isolates tested here, except H6, we indeed observed adverse effects on the salmon eggs (S4B Fig). Further studies should also look into effects of *P*. *fluorescens* H6 on the life-cycle of salmon post hatching.

## Conclusions

Aquaculture is one of the fastest growing animal food sectors [45], partly as a response to the increasing demand for fish protein and regulations to prevent overfishing from wild populations [7]. Considering the long-term importance of aquaculture for food production and economic development, sustainable measures are urgently needed to mitigate emerging diseases including Saprolegniosis. Although the biosurfactant from *Pseudomonas* H6 did not show activity *in vivo*, the bacterial strain itself did provide promising antagonistic activity against *Saprolegnia* infections of salmon eggs. Our research provides a framework for selecting beneficial bacteria that can suppress Saprolegniosis and possibly other emerging diseases in aquaculture.

## Supporting Information

S1 Fig Overall strategy used to decipher diversity of aquatic *Pseudomonas* species and their activity against the fish pathogenic oomycete *Saprolegnia*. (TIF)

S2 Fig Colony count of salmon egg incubation water dilution-plated on 1/10TSA and PSA. Error bars represent S.E.M. (*N* = 6).
(TIF)

S3 Fig *In vivo* activity of the 11 aquatic *Pseudomonas* strains against *S*. *diclina* on salmon eggs. The mean percentage of egg mortality caused by *S*. *diclina* 765F3 was determined at 6 days post inoculation (dpi) of this oomycete pathogen. *Pseudomonas* strains D1-D3 and S1 originated from diseased salmon eggs, whereas strains S2 and H1-H6 originated from healthy salmon eggs. All strains were introduced at an initial cell density of 10^8^ CFU ml^-1^. The incubation temperature was 10±1°C. Error bars represent S.E.M. (*N* = 3). The asterisk indicates a statistically significant difference from the control (*S*. *diclina* only) based on a one-way analysis of variance and *post hoc* LSD analysis (*P*<0.05).
(TIF)

S4 Fig *In vivo* activity of 11 *Pseudomonas* isolates against *S*. *parasitica* and their effect on salmon egg mortality. In bioassay 1, the initial bacterial density was 10^8^ CFU ml^-1^ and incubation temperature was 10±1°C. (A) Mean percentage of egg mortality was determined at 6 dpi of *S*. *parasitica* 762F4 [22]. *Pseudomonas* H6 reduced (*P* = 0.062) mortality compared to the control with *S*. *parasitica* only. (B) Mean percentage of egg mortality inoculated with cell suspensions of 11 *Pseudomonas* strains only. Error bars represent S.E.M. (*N* = 3). Asterisks indicate statistically significant differences from the controls, *S*. *parasitica* only (A) or non-treated (B) based on one-way analysis of variance and *post hoc* LSD analysis on ArcSin square root transformed data (*P*<0.05).
(TIF)

S1 Table BOX-PCR genotypic grouping of bacteria isolated from diseased and healthy salmon eggs by plating incubation water on the *Pseudomonas* semi-selective medium PSA. Only the BOX groups that consisted of at least 4 isolates from either diseased or healthy salmon egg samples are shown. One representative isolate from each BOX group was selected for activity testing in salmon egg bioassays. From the shared representative isolates, *Pseudomonas* isolates S1 and S2, which belonged to the largest shared BOX group and originated from both healthy and diseased salmon eggs were selected.^a^ Isolates H3 and S2 were obtained from 1/10TSA, not from PSA.
(PDF)

S2 Table Effect of biosurfactants from *Pseudomonas* H6 and *P*. *fluorescens* SS101 on hyphal diameter of *S*. *parasitica* CBS 223.65. Diameter of 10 *S*. *parasitica* hyphae of each treatment was measured by ImageJ 1.47v. Mean diameter and standard error of the mean are shown. Asterisks indicate statistically significant differences compared to the controls, based on a one-way analysis of variance and *post hoc* LSD analysis (*P*<0.05).
(PDF)

## References

[1] Fisher MC, Henk DA, Briggs CJ, Brownstein JS, Madoff LC, McCraw SL, et al Emerging fungal threats to animal, plant and ecosystem health. Nature. 2012;484(7393):186–94. http://www.nature.com/nature/journal/v484/n7393/abs/nature10947.html#supplementary-information. 10.1038/nature10947 22498624 PMC3821985

[2] Gozlan RE, Marshall W, Lilje O, Jessop C, Gleason FH, Andreou D. Current ecological understanding of fungal-like pathogens of fish: what lies beneath? Frontiers in Microbiology. 2014;5 10.3389/fmicb.2014.00062 PMC3928546 24600442

[3] Sarmiento-Ramírez JM, Abella-Pérez E, Phillott AD, Sim J, van West P, Martín MP, et al Global Distribution of Two Fungal Pathogens Threatening Endangered Sea Turtles. PLoS ONE. 2014;9(1):e85853 10.1371/journal.pone.0085853 24465748 PMC3897526

[4] Martel A, Spitzen-van der Sluijs A, Blooi M, Bert W, Ducatelle R, Fisher MC, et al *Batrachochytrium salamandrivorans* sp. nov. causes lethal chytridiomycosis in amphibians. Proceedings of the National Academy of Sciences of the United States of America. 2013;110(38):15325–9. 10.1073/pnas.1307356110 . 24003137 PMC3780879

[5] Woodhams DC, Bosch J, Briggs CJ, Cashins S, Davis LR, Lauer A, et al Mitigating amphibian disease: strategies to maintain wild populations and control chytridiomycosis. Frontiers in Zoology. 2011;8 10.1186/1742-9994-8-8 . PMC3098159 21496358

[6] Phillips AJ, Anderson VL, Robertson EJ, Secombes CJ, van West P. New insights into animal pathogenic oomycetes. Trends in Microbiology. 2008;16(1):13–9. 10.1016/j.tim.2007.10.013 18096392

[7] Bruno D, van West P, Beakes G. *Saprolegnia* and other oomycetes In: Woo P, Bruno D, editors. Fish Diseases and Disorders, Viral, Bacterial and Fungal Infections. 3 2nd ed. Wallingford, UK: CABI: Wallingford, UK; 2011 p. 669–720.

[8] van den Berg AH, McLaggan D, Diéguez-Uribeondo J, van West P. The impact of the water moulds *Saprolegnia diclina* and *Saprolegnia parasitica* on natural ecosystems and the aquaculture industry. Fungal Biology Reviews. 2013;27(2):33–42. 10.1016/j.fbr.2013.05.001

[9] Fernández-Benéitez MJ, Ortiz-Santaliestra ME, Lizana M, Diéguez-Uribeondo J. *Saprolegnia diclina*: another species responsible for the emergent disease ‘*Saprolegnia* infections’ in amphibians. FEMS Microbiology Letters. 2008;279(1):23–9. 10.1111/j.1574-6968.2007.01002.x 18177304

[10] Krugner-Higby L, Haak D, Johnson P, Shields J, Jones WI, Reece K, et al Ulcerative disease outbreak in crayfish *Orconectes propinquus* linked to *Saprolegnia australis* in Big Muskellunge Lake, Wisconsin. Diseases of Aquatic Organisms. 2010;91(1):57–66. 10.3354/dao02237 20853742

[11] van West P. *Saprolegnia parasitica*, an oomycete pathogen with a fishy appetite: new challenges for an old problem. Mycologist. 2006;20(3):99–104. 10.1016/j.mycol.2006.06.004

[12] Das SK, Murmu K, Das A, Shakuntala I, Das RK, Ngachan SV, et al Studies on the identification and control of pathogen *Saprolegnia* in selected Indian major carp fingerlings at mid hill altitude. Journal of environmental biology / Academy of Environmental Biology, India. 2012;33(3):545–9. Epub 2012/10/04. . 23029901

[13] Hatai K, Hoshiai G. Mass mortality in cultured coho salmon (*Oncorhynchus kisutch*) due to *Saprolegnia parasitica* coker. Journal of Wildlife Diseases. 1992;28(4):532–6. 10.7589/0090-3558-28.4.532 1474649

[14] Hatai K, Hoshiai G-I. Pathogenicity of *Saprolegnia parasitica* Coker In: Mueller GJ, editor. Salmon Saprolegniasis. Portland, Oregon: U.S. Department of Energy, Bonneville Power Administration, Portland, Oregon; 1994.

[15] Lategan MJ, Gibson LF. Antagonistic activity of *Aeromonas media* strain A199 against *Saprolegnia* sp., an opportunistic pathogen of the eel, *Anguilla australis* Richardson. Journal of Fish Diseases. 2003;26(3):147–53. 10.1046/j.1365-2761.2003.00443.x . 12962224

[16] Lategan MJ, Torpy FR, Gibson LF. Biocontrol of saprolegniosis in silver perch *Bidyanus bidyanus* (Mitchell) by *Aeromonas media* strain A199. Aquaculture. 2004;235(1–4):77–88. 10.1016/j.aquaculture.2003.09.014 .

[17] Lategan MJ, Torpy FR, Gibson LF. Control of saprolegniosis in the eel *Anguilla australis* Richardson, by *Aeromonas media* strain A199. Aquaculture. 2004;240(1–4):19–27. 10.1016/j.aquaculture.2004.04.009 .

[18] Hatai K, Willoughby LG. *Saprolegnia parasitica* from rainbow trout inhibited by the bacterium *Pseudomonas fluorescens* . Bull Eur Ass Fish Pathol. 1988;8(2):27–9.

[19] Hussein MMA, Hatai K. *In vitro* inhibition of *Saprolegnia* by bacteria isolated from lesions of salmonids with saprolegniasis. Fish Pathology. 2001;36(2):73–8. .

[20] Bly JE, Quiniou SMA, Lawson LA, Clem LW. Inhibition of *Saprolegnia* pathogenic for fish by *Pseudomonas fluorescens* . Journal of Fish Diseases. 1997;20(1):35–40. 10.1046/j.1365-2761.1997.d01-104.x .

[21] Carbajal-González MT, Fregeneda-Grandes JM, Suárez-Ramos S, Rodríguez-Cadenas F, Aller-Gancedo JM. Bacterial skin flora variation and in vitro inhibitory activity against *Saprolegnia parasitica* in brown and rainbow trout. Diseases of Aquatic Organisms. 2011;96(2):125–35. 10.3354/dao02391 . 22013752

[22] Liu Y, de Bruijn I, Jack ALH, Drynan K, van den Berg AH, Thoen E, et al Deciphering microbial landscapes of fish eggs to mitigate emerging diseases. ISME J. 2014;8(10):2002–14. 10.1038/ismej.2014.44 24671087 PMC4184010

[23] de Bruijn I, de Kock MJD, Yang M, de Waard P, van Beek TA, Raaijmakers JM. Genome-based discovery, structure prediction and functional analysis of cyclic lipopeptide antibiotics in *Pseudomonas* species. Molecular Microbiology. 2007;63(2):417–28. 10.1111/j.1365-2958.2006.05525.x 17241198

[24] De Souza JT, De Boer M, De Waard P, Van Beek TA, Raaijmakers JM. Biochemical, genetic, and zoosporicidal properties of cyclic lipopeptide surfactants produced by *Pseudomonas fluorescens* . Applied and environmental microbiology. 2003;69(12):7161–72. Epub 2003/12/09. ; PubMed Central PMCID: PMCPmc309978. 14660362 10.1128/AEM.69.12.7161-7172.2003 PMC309978

[25] Raaijmakers JM, de Bruijn I, de Kock MJD. Cyclic lipopeptide production by plant-associated *Pseudomonas* spp.: diversity, activity, biosynthesis, and regulation. Molecular Plant-Microbe Interactions. 2006;19(7):699–710. 10.1094/MPMI-19-0699 16838783

[26] Rademaker JLW, Louws FJ, de Bruijn FJ. Characterization of the diversity of ecologically important microbes by rep-PCR genomic fingerprinting In: Akkermans ADL, van Elsas JD, de Bruijn FJ, editors. Molecular Microbial Ecology Manual. Dordrecht: Kluwer; 1998 p. 1–26.

[27] Versalovic J, Schneider M, de Bruijn FJ, Lupski JR. Genomic fingerprinting of bacteria using repetitive sequence based PCR (rep-PCR). Meth Cell Mol Biol. 1994;5:25–40.

[28] Loper JE, Hassan KA, Mavrodi DV, Davis EW II, Lim CK, Shaffer BT, et al Comparative genomics of plant-associated *Pseudomonas* spp.: insights into diversity and inheritance of traits involved in multitrophic interactions. PLoS Genet. 2012;8(7):e1002784 10.1371/journal.pgen.1002784 22792073 PMC3390384

[29] Kimura M. A simple method for estimating evolutionary rates of base substitutions through comparative studies of nucleotide sequences. J Mol Evol. 1980;16(2):111–20. Epub 1980/12/01. . 7463489 10.1007/BF01731581

[30] Nguyen DD, Wu C-H, Moree WJ, Lamsa A, Medema MH, Zhao X, et al MS/MS networking guided analysis of molecule and gene cluster families. Proceedings of the National Academy of Sciences. 2013 10.1073/pnas.1303471110 PMC3710860 23798442

[31] Watrous J, Roach P, Alexandrov T, Heath BS, Yang JY, Kersten RD, et al Mass spectral molecular networking of living microbial colonies. Proceedings of the National Academy of Sciences. 2012 10.1073/pnas.1203689109 PMC3387089 22586093

[32] Pierce CY, Barr JR, Cody RB, Massung RF, Woolfitt AR, Moura H, et al Ambient generation of fatty acid methyl ester ions from bacterial whole cells by direct analysis in real time (DART) mass spectrometry. Chemical Communications. 2007;(8):807–9. 10.1039/B613200F 17308638

[33] Cheng X, van der Voort M, Raaijmakers JM. Gac-mediated changes in pyrroloquinoline quinone biosynthesis enhance the antimicrobial activity of *Pseudomonas fluorescens* SBW25. Environmental Microbiology Reports. 2015:n/a-n/a. 10.1111/1758-2229.12231 25356880

[34] Schneider CA, Rasband WS, Eliceiri KW. NIH Image to ImageJ: 25 years of image analysis. Nat Meth. 2012;9(7):671–5. 10.1038/nmeth.2089 PMC5554542 22930834

[35] Verschuere L, Rombaut G, Sorgeloos P, Verstraete W. Probiotic bacteria as biological control agents in aquaculture. Microbiology and Molecular Biology Reviews. 2000;64(4):655–+. 10.1128/mmbr.64.4.655-671.2000 . 11104813 PMC99008

[36] Garces ME, Sequeiros C, Olivera NL. Marine *Lactobacillus pentosus* H16 protects *Artemia franciscana* from *Vibrio alginolyticus* pathogenic effects. Diseases of Aquatic Organisms. 2015;113(1):41–50. 10.3354/dao02815 . 25667335

[37] de Bruijn I, de Kock MJD, de Waard P, van Beek TA, Raaijmakers JM. Massetolide a biosynthesis in *Pseudomonas fluorescens* . Journal of Bacteriology. 2008;190(8):2777–89. 10.1128/jb.01563-07 . 17993540 PMC2293227

[38] van de Mortel JE, Ha T, Govers F, Raaijmakers JM. Cellular Responses of the Late Blight Pathogen *Phytophthora infestans* to Cyclic Lipopeptide Surfactants and Their Dependence on G Proteins. Applied and environmental microbiology. 2009;75(15):4950–7. 10.1128/aem.00241-09 . 19502443 PMC2725516

[39] Nielsen TH, Sørensen J. Production of cyclic lipopeptides by *Pseudomonas fluorescens* strains in bulk soil and in the sugar beet rhizosphere. Applied and environmental microbiology. 2003;69(2):861–8. 10.1128/aem.69.2.861-868.2003 . 12571005 PMC143599

[40] Abd-Allah AMA, Srorr T. Biodegradation of anionic surfactants in the presence of organic contaminants. Water Research. 1998;32(3):944–7. 10.1016/S0043-1354(97)00223-6

[41] Abu-Ghunmi L, Badawi M, Fayyad M. Fate of Triton X-100 Applications on Water and Soil Environments: A Review. J Surfact Deterg. 2014;17(5):833–8. 10.1007/s11743-014-1584-3

[42] Song C, Aundy K, van de Mortel J, Raaijmakers JM. Discovery of new regulatory genes of lipopeptide biosynthesis in *Pseudomonas fluorescens* . FEMS Microbiology Letters. 2014;356(2):166–75. 10.1111/1574-6968.12404 25202778

[43] Austin B, Austin DA. Bacterial fish pathogens: disease of farmed and wild fish: Springer Science & Business Media; 2007.

[44] Loch TP, Scribner K, Tempelman R, Whelan G, Faisal M. Bacterial infections of Chinook salmon, *Oncorhynchus tshawytscha* (Walbaum), returning to gamete collecting weirs in Michigan. J Fish Dis. 2012;35(1):39–50. Epub 2011/12/16. 10.1111/j.1365-2761.2011.01322.x . 22168454

[45] FAO. The state of world fisheries and aquaculture 2014. Rome, Italy2014. 223 p.

