# Supplementary material for: Diversity of Aquatic Pseudomonas Species and Their Activity against the Fish Pathogenic Oomycete Saprolegnia
Source: PLoS One. 2015 Aug 28;10(8):e0136241. doi: 10.1371/journal.pone.0136241 (PMC4552890; doi:10.1371/journal.pone.0136241)
Supplement: S1 Table — Only the BOX groups that consisted of at least 4 isolates from either diseased or healthy salmon egg samples are shown. One representative isolate from each BOX group was selected for activity testing in salmon egg bioassays. From the shared representative isolates, Pseudomonas isolates S1 and S2, which belonged to the largest shared BOX group and originated from both healthy and diseased salmon eggs were selected.a Isolates H3 and S2 were obtained from 1/10TSA, not from PSA. (PDF) [file pone.0136241.s005.pdf]

| BOX groups | No. of isolates | | Isolates selected for phylogeny analysis | Positive against *Saproelgnia* hyphal growth | Capable of producing biosurfactants | Isolates selected for testing in salmon egg bioassays |
|---|---|---|---|---|---|---|
| | Diseased | Healthy | | | | |
| 1 | 9 | 0 | D1 | Yes | No | D1 |
| 2 | 7 | 0 | D2 | Yes | Yes | D2 |
| 3 | 6 | 0 | D3 | Yes | Yes | D3 |
| 4 | 0 | 23 | H1 | Yes | Yes | H1 |
| 5 | 0 | 7 | H2 | Yes | Yes | H2 |
| 6 | 0 | 5 | H3[a] | Yes | Yes | H3[a] |
| 7 | 0 | 5 | H4 | Yes | Yes | H4 |
| 8 | 0 | 4 | H5 | Yes | Yes | H5 |
| 9 | 0 | 4 | H6 | Yes | Yes | H6 |
| 10 | 30 | 15 | S1, S2[a] | Yes | No | S1, S2[a] |
| 11 | 10 | 9 | S3, S4 | Yes | No | |
| 12 | 11 | 7 | S5, S6 | Yes | Yes | |
| 13 | 10 | 4 | S7, S8 | Yes | Yes | |
| 14 | 10 | 1 | S9, S10 | Yes | No | |
| 15 | 3 | 8 | S11, S12 | Yes | S11: No; S12: Yes | |
| 16 | 7 | 1 | S13, S14 | Yes | Yes | |
| 17 | 5 | 1 | S15, S16 | Yes | No | |
| 18 | 2 | 4 | S17, S18 | Yes | No | |
