# Supplementary material for: Diversity of Aquatic Pseudomonas Species and Their Activity against the Fish Pathogenic Oomycete Saprolegnia
Source: PLoS One. 2015 Aug 28;10(8):e0136241. doi: 10.1371/journal.pone.0136241 (PMC4552890; doi:10.1371/journal.pone.0136241)
Supplement: S2 Table — Diameter of 10 S. parasitica hyphae of each treatment was measured by ImageJ 1.47v. Mean diameter and standard error of the mean are shown. Asterisks indicate statistically significant differences compared to the controls, based on a one-way analysis of variance and post hoc LSD analysis (P<0.05). (PDF) [file pone.0136241.s006.pdf]

		Control	15	40	100	200	($\mu g\ ml^{-1}$)
Hyphal	H6	0.009±0.0005	0.012±0.0007*	0.014±0.0008*	0.017±0.0014*	0.017±0.0008*	(mm)
thickness	SS101	0.007±0.0006	0.011±0.0008*	0.013±0.0007*	0.011±0.0008*	0.013±0.0007*	(mm)
